# Risk Factors for Community-Acquired Urinary Tract Infections Caused by Multidrug-Resistant Enterobacterales in Thailand

**DOI:** 10.3390/antibiotics11081039

**Published:** 2022-08-02

**Authors:** Kanit Assawatheptawee, Pornpit Treebupachatsakul, Taradon Luangtongkum, Pannika R. Niumsup

**Affiliations:** 1 Department of Microbiology and Parasitology, Faculty of Medical Science, Naresuan University, Phitsanulok 65000, Thailand; kanit_as@outlook.com; 2 Buddhachinnaraj Hospital, Phitsanulok 65000, Thailand; pornpitt@gmail.com; 3 Department of Veterinary Public Health, Faculty of Veterinary Science, Chulalongkorn University, Bangkok 10330, Thailand; taradon.l@chula.ac.th; 4 Center of Excellent in Medical Biotechnology, Faculty of Medical Science, Naresuan University, Phitsanulok 65000, Thailand

**Keywords:** community, urinary tract infection, multidrug resistance, Enterobacterales, risk factors, *mcr-9*

## Abstract

The dissemination of multidrug-resistant Enterobacterales (MDRE) in community settings is becoming a great concern. This study aimed to assess the incidence and risk factors associated with community-acquired urinary tract infections (CA-UTIs) caused by MDRE. A prospective case–control study was undertaken among patients with UTIs visiting an outpatient department in Phitsanulok Province, Thailand. Urine samples were collected and screened to include only patients with Enterobacterales infections. Risk factors were analyzed by multivariate logistic regression analysis. Of the 284 patients with CA-UTIs, 25.7% (*n* = 73) and 74.3% (*n* = 211) were positive for MDRE (case) and non-MDRE (control), respectively. Being a farmer was identified as an independent risk factor for MDRE-associated CA-UTIs (adjusted odds ratio = 3.101; 95% confidence interval = 1.272–7.564; *p* = 0.013). A total of 309 Enterobacterales isolates were recovered, and *Escherichia coli* was the most frequently detected (86.4%). The highest resistance rate was observed for ampicillin (67.0%), followed by ciprofloxacin (34.0%) and cotrimoxazole (32.7%), while resistance to third-generation cephalosporins (cefotaxime, ceftriaxone) and levofloxacin remained <20%. Resistance to ampicillin–gentamicin–cotrimoxazole was the most common pattern among MDRE isolates. Interestingly, we detected a colistin-resistant *Enterobacter cloacae* harboring *mcr-9* (colistin MIC = 16 µg/mL). *mcr-9* was transferable at high frequency (4.5 × 10^−4^) and resided on IncF plasmid. This study demonstrates that being a farmer is a risk factor for MDRE-associated CA-UTIs. Interestingly, this is the first report to identify *mcr-9*-positive *E. cloacae* from a Thai patient in the community.

## 1. Introduction

Urinary tract infections are common bacterial infections in hospital and community settings. Community-acquired urinary tract infections (CA-UTIs) affect people of all ages, although the incidence rates are higher in women. A UTI usually begins with urethral contamination by bacteria residing in the intestine that subsequently spread to the bladder. UTIs can be asymptomatic or symptomatic, ranging from mild to life-threatening infections, if left untreated [1]. It has been shown that in 27% of patients with septicemia presenting to an emergency department, the septicemia resulted from previous UTIs [2]. Enterobacterales are common etiological agents of CA-UTIs. Of these, *Escherichia coli* is the predominant causative organism, accounting for more than 80% of infections. Other organisms include *Enterococcus* spp., *Staphylococcus aureus,* and *Proteus mirabilis* [1].

In poor-resource settings, UTIs were frequently managed by empirical antibiotic therapy before urine cultures and susceptibility data were available. This practice undoubtedly led to an increase in the prevalence of antibiotic-resistant bacteria. Due to the variety of causative agents and antibiotic resistance patterns between regions, empiric therapy for UTIs is, presently, based on local susceptibility data. However, a recent systematic review showed that in many countries in the Asia-Pacific (APAC) region, empirical treatment was given to patients with UTIs without national antimicrobial resistance data [3]. In accordance with this report, a study on uncomplicated UTIs among outpatients in central Thailand demonstrated a very high prevalence (91.3%) of inappropriate empirical therapy [4]. Broad-spectrum antibiotics, such as cotrimoxazole, nitrofurantoin, fosfomycin, β-lactam, and fluoroquinolone, have been used as an empirical treatment to treat UTIs [5]. However, treatment is becoming more difficult owing to the rapid spread of antibiotic resistance among Enterobacterales.

Multidrug resistance (MDR) is one of the greatest challenges for public health. Diseases caused by MDR bacteria result in a prolonged hospital length of stay, high mortality rate, and increased treatment cost. A previous study in northeastern Thailand showed that the 30-day mortality in Thai patients with MDR community-acquired bacteremia was as high as 35% [6]. The average treatment cost per Thai patient increased by 42% in drug-resistant infections compared to that in drug-susceptible cases. Furthermore, the annual treatment and antibiotic costs associated with the management of drug-resistant infections were estimated to be up to USD 2.3 billion and USD 262 million, respectively [7]. Alternative methods for controlling drug-resistant bacteria have been reported with a significant reduction in the duration of antibiotic therapy. This could avoid the overuse of antibiotics and, consequently, antibiotic resistance [8]. 

Southeast Asia has been dealing with a high prevalence of MDR Enterobacterales (MDRE) for a long time [9]. In Thailand, one of the driving forces of the rapid spread of MDRE in community settings is due to the fact that people are usually self-medicated because antibiotics are freely accessible in community pharmacies [10]. Data on susceptibility profiles of *E. coli* causing CA-UTIs in the APAC countries in 2012 revealed the relatively high rates of resistance to third-generation cephalosporins (25.6–34.9%) and fluoroquinolones (43.1–44.4%) [11]. According to the National Antimicrobial Resistance Surveillance Center, Thailand, the rates of resistance to cotrimoxazole, ciprofloxacin, and ceftriaxone among *E. coli* isolates from patients with CA-UTIs from 83 hospitals across the country in 2020 (*n* = 5851) were 56.7%, 66.6%, and 39.8%, respectively [12]. 

Cases of CA-UTIs caused by MDRE continue to rise around the world. Early identification of patients at risk for MDRE-associated CA-UTIs may help to guide the appropriate empirical treatment [13,14]. Delays in initiating empirical therapy may lead to severe diseases and high treatment-related costs. In Thailand, most studies on CA-UTIs have concentrated on ESBL-producing Enterobacterales. Many studies demonstrated that *E. coli* was the most common causative agent of CA-UTIs in both children and adults. Kidney disease was identified as an associated risk factor for CA-UTIs caused by ESBL-producing bacteria in children, while in adult patients, previous UTIs with ESBL-producing *E. coli* and prior use of antibiotics (cephalosporin and penicillin) were identified as independent risk factors. Furthermore, appropriate antibiotic therapy was delayed for patients with CA-UTIs due to ESBL-producing bacteria [15,16,17]. 

The dissemination of MDR bacteria in community settings is becoming a great concern because it is more difficult to control than in hospitals. In Thailand, data on MDRE-associated CA-UTIs are not well defined. With the increasing prevalence of MDRE, reliable predictors of CA-UTIs caused by MDRE and susceptibility data are needed. This study was carried out to investigate the incidence and risk factors associated with CA-UTIs caused by MDRE. Characterization of MDRE isolates was also carried out. 

## 2. Results

### 2.1. Patient Characteristics and Isolation of MDRE from Patients with CA-UTIs

**Figure 1:**
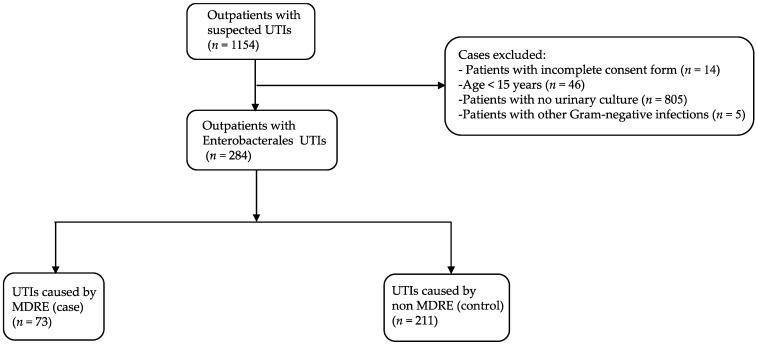
Flowchart of patients included in this study. UTI, urinary tract infection; MDRE, multidrug-resistant Enterobacterales.

During the study period, 1154 patients, who visited an outpatient department with a suspected UTI and had submitted a urine sample for culture, agreed to participate in this study. A total of 284 patients who had CA-UTIs caused by Enterobacterales were included for further analysis (Figure 1). The median age of participants was 44.5 years (range, 15–99 years). The patients’ characteristics are shown in Table 1. The majority were female (88.4%), lived in an urban area (56.7%), had a secondary school education (40.5%), and worked as a government officer or company employee (24.3%). Hypertension was the most common underlying disease (21.1%). Prior antibiotic use within the last 3 months was observed in 32 patients (11.3%), and only 17 patients (6.0%) had a history of hospitalization within the previous 6 months. 

Among 284 urine samples that yielded Enterobacterales, 25.7% (*n* = 73) and 74.3% (*n* = 211) were positive for MDRE (case) and non-MDRE (control), respectively (Figure 1). A total of 309 non-duplicated Enterobacterales isolates were recovered (Table 2). The most common organism was *E. coli* (*n* = 267), accounting for 86.4% of isolates, followed by *Klebsiella pneumoniae* (*n* = 22, 7.1%), *Enterobacter cloacae* (*n* = 11, 3.6%) and *Proteus* spp. (*n* = 4, 1.3%). Other Enterobacterales isolates, including *Enterobacter aerogenes*, *Proteus mirabilis*, *Citrobacter* spp., and *Salmonella* spp., were detected at low frequencies (1–2 isolates each) (Table 2).

### 2.2. Risk Factors for CA-UTIs Caused by MDRE

Risk factors associated with CA-UTIs were analyzed by comparing patients with and without MDRE (Table 1). According to univariate analysis and multivariate logistic regression analysis, being a farmer (*p* = 0.013, aOR 3.101, 95% CI = 1.272–7.564) was identified as an independent risk factor for CA-UTIs caused by MDRE.

### 2.3. Susceptibility Test for Enterobacterales

The susceptibility test was performed on all 309 Enterobacterales isolates (77 MDRE and 232 non-MDRE) (Table 3). The highest resistance rate was observed for ampicillin (67.0%), followed by ciprofloxacin (34.0%) and cotrimoxazole (32.7%). Rates of resistance to cephalexin, cefuroxime, cefotaxime, ceftriaxone, gentamicin, and levofloxacin were 13.6–19.7%, while lower rates of resistance to amoxicillin/clavulanate, amikacin, fosfomycin, and nitrofurantoin were observed (1.3–6.1%). All isolates were susceptible to imipenem. Resistance rates for MDRE were significantly higher (*p* < 0.001) than non-MDRE for several antibiotics tested, except amoxicillin/clavulanate, fosfomycin, and nitrofurantoin. As many as 32 resistance patterns among MDRE and 36 among non-MDRE were observed (Tables S1 and S2). Resistance to ampicillin–gentamicin–cotrimoxazole was the most common pattern in MDRE (11.7%), while resistance to ampicillin alone was frequently found in non-MDRE (20.3%) (Table 4).

All isolates were tested for resistance to colistin by the broth microdilution method. All but one isolates demonstrated colistin MICs of 0.5–1 µg/mL. A single isolate, identified as *Enterobacter cloacae*, showed a colistin MIC of 16 µg/mL.

### 2.4. Characterization of the Colistin-Resistant Isolate

Further identification of the resistant determinant for colistin resistance in *E.cloacae* revealed that this isolate carried *mcr-9*. No other *mcr* was detected. *E. cloacae* was able to transfer *mcr-9* to the recipient, *E. coli* J53, with a frequency of 4.5 × 10^−4^ transconjugants per donor cell. Following conjugation, transconjugant carrying *mcr-9* showed a fourfold increase in colistin MIC (2 µg/mL) compared to the recipient, *E. coli* J53 (0.5 µg/mL). Plasmid-based replicon typing showed that *mcr-9* resided on the IncF plasmid.

## 3. Discussion

This prospective case–control study investigated the incidence and risk factors associated with CA-UTIs caused by MDRE among patients visiting an outpatient department in lower northern Thailand. Of the 284 patients with CA-UTIs, we noted that the incidence of CA-UTIs caused by MDRE was 25.7%. This result was higher than the incidence rates reported in the USA (12–19%) [18,19] but was lower than that in Tunisia (45.1%) [20]. These differences may be partly attributed to variations in the study designs, settings, and participants. 

With respect to risk factors, several reports, mostly retrospective studies, revealed that age >65 or ≥70 years, residing in a nursing home, presence of diabetes mellitus, presence of obstructive uropathy, history of urinary tract surgery, previous antibiotic usage, and prior hospitalization were independent risk factors for MDRE-associated CA-UTIs [19,20,21]. Another study in the USA showed that frequent consumption of chicken was associated with CA-UTIs caused by MDR *E. coli* [18]. The independent risk factors for MDRE-associated CA-UTIs identified in our study differed markedly from those reported in previous literature. We found that antibiotic usage within the previous 3 months was significantly higher in the MDRE group (*p* = 0.04); however, this was not identified as a risk factor in the final multivariate logistic regression analysis (*p* = 0.062) (Table 1). Interestingly, we demonstrated that being a farmer was an independent risk factor for MDRE-associated CA-UTIs (*p* = 0.013, aOR = 3.101). This might be explained by the fact that farmers are frequently exposed to animals and environments that are contaminated with MDRE [22]. Consequently, farmers may become asymptomatic carriers of MDRE, mostly in their intestinal tract. Then, MDRE can spread to the urinary tract, where it can multiply and cause UTIs [1]. This explanation is supported by a previous study showing that uropathogens are often found in the intestine of the same host [23]. In addition, the transmission of *E. coli* from anus to urethra has been genetically demonstrated among Thai patients with CA-UTIs [24]. 

The most frequently detected species from CA-UTIs was *E. coli*, accounting for 86.4% of all isolates. High resistance rates were observed for ampicillin, ciprofloxacin, and cotrimoxazole (32.7–67.0%), while resistance to third-generation cephalosporins (cefotaxime, ceftriaxone) and levofloxacin remained <20%. Compared with previous studies on CA-UTIs caused by Enterobacterales in central Thailand, our study shows that rates of resistance to cotrimoxazole, third-generation cephalosporins, and ciprofloxacin were lower, 32.7% vs. ~41–60%, ~14% vs. ~20–22% and 34.0% vs. 42–49%, respectively [4,17].

Ciprofloxacin and cotrimoxazole are commonly used as empirical treatments for UTIs. According to the US and European guidelines, cotrimoxazole should not be used if the resistance rate exceeds 20%, and ciprofloxacin may be used when there are no other alternative drugs. In addition, the European guidelines suggest that fluoroquinolones may be used if the resistance rates are less than 10% [5,25]. Our study revealed that the rates of resistance to ciprofloxacin and cotrimoxazole were 34.0% and 32.7%, respectively, suggesting that the use of both drugs should be avoided. In fact, fluoroquinolones have been shown to be inappropriate as empirical therapy for treating CA-UTIs in APAC countries [11]. Amoxicillin/clavulanate, amikacin, fosfomycin, and nitrofurantoin may be considered reasonable treatment options for CA-UTIs since resistance was seen in only a limited number of isolates (Table 3). 

Colistin is one of the few last-resort antibiotics for the treatment of serious infections caused by MDRE. In the past, colistin resistance in Enterobacterales was not common and mainly arose from chromosomal mutations. In 2016, a transferable colistin resistance determinant, mobile colistin resistance (*mcr*), was found in an *E. coli* isolate from a pig [26]. Subsequently, several *mcr* variants (*mcr-1*–*mcr-10*) were reported from different countries and with different sample origins. Of these, *mcr-1* and *mcr-9* are the most widely disseminated [27]. In Thailand, many studies have documented the presence of *mcr*-positive Enterobacterales in humans and animals, mostly *mcr-1* followed by *mcr-3* [28,29]. Interestingly, we obtained colistin-resistant *E. cloacae* isolate that carried the transferable *mcr-9*. Nowadays, *mcr-9* is mostly found in humans [30]. In Thailand, however, only a single study demonstrated the presence of *mcr-9* in *E. coli* isolates from slaughtered pigs [31]. The detection of *mcr-9*-positive *E. cloacae* in our study corresponds to the fact that *Enterobacter* spp. are among the most common host species for *mcr-9* [30]. Our *mcr-9* was found to reside on IncF plasmid, in contrast to the published literature, which suggested that the global spread of *mcr-9* appears to be driven by a plasmid with IncHI2 replicon type [30]. Although IncF is a narrow host range plasmid, it could transfer resistance genes to several species in Enterobacterales [32]. 

In conclusion, this study demonstrates that being a farmer is a risk factor for CA-UTIs caused by MDRE. Our data on susceptibility profiles may be useful in selecting appropriate empirical antibiotic therapy for MDRE-associated CA-UTIs. Interestingly, this is the first identification of *mcr-9*-positive colistin-resistant *E. cloacae* from a patient in a community in Thailand, which is alarming and requires more serious attention. Further investigations are necessary to provide a better understanding of its dissemination within the community.

## 4. Materials and Methods

### 4.1. Study Setting and Patients

We conducted a prospective case-control study on CA-UTIs at 1000-bed tertiary care teaching hospital in Phitsanulok Province, lower northern Thailand, from April 2018 to October 2020. Patients, who were ≥15 years old, had visited an outpatient department and were suspected of having a UTI based on signs and symptoms (dysuria, frequency, urgency, and suprapubic pain) and pyuria (>10 white blood cells per high-power field) were eligible for this study. Re-visiting of the same patient and patients who were <15 years old were excluded. Urine samples were collected and then evaluated to include only patients with cultures positive for Enterobacterales. The study was approved by the Naresuan University Review Board (COA No. 178/2018 and 011/2021). Written informed consent was obtained from patients prior to study participation. 

### 4.2. Data Collection

Patients were asked to fill in a structured questionnaire to collect information regarding demographic data (age, gender, education, occupation, place of residence, etc.), pet ownership, raising backyard poultry, underlying conditions, consumption of undercooked meat, antibiotic usage within previous 3 months and history of hospitalization within previous 6 months.

### 4.3. Microbiological Methods

Midstream urine specimens were collected using sterile screw-capped containers. Approximately 20 mL of urine was collected from each subject. Urine samples were analyzed for the presence of Enterobacterales by inoculating 1 mL into 9 mL Enterobacteriaceae Enrichment broth (Oxoid Ltd, Basingstoke, UK) and incubating at 37 °C overnight. Then, one loopful of overnight culture was streaked on MacConkey agar (Oxoid Ltd) and incubated at 37 °C for 24 h. Colonies of different morphologies were picked and species identification was performed by biochemical tests using API 20E (bioMérieux SA, Marcy-l’Etoile, France). Suspected colonies were further verified by 16S rDNA sequencing [33]. 

All Enterobacterales isolates were tested for susceptibility to 14 antimicrobial agents by disk diffusion method using the interpretive breakpoints recommended by the Clinical and Laboratory Standards Institute [34]. The antibiotics tested (Oxoid Ltd) included ampicillin, amoxicillin/clavulanate, cephalexin, cefuroxime, cefotaxime, ceftriaxone, imipenem, gentamicin, amikacin, cotrimoxazole, ciprofloxacin, levofloxacin, fosfomycin, and nitrofurantoin. Isolates showing intermediate results were considered to be susceptible. *E. coli* DMST4212, obtained from the Department of Medical Sciences, Ministry of Public Health, Bangkok, was used as a control strain. Colistin minimum inhibitory concentrations (MICs) were determined by broth microdilution method according to CLSI guidelines [34]. CLSI breakpoints were used for interpreting colistin MICs (resistance, ≥4 µg/mL).

### 4.4. Definitions

MDRE was defined as isolates showing resistance to at least 3 antibiotic classes [35]. Patients were assigned as cases if they had a positive urine culture for MDRE, while patients with non-MDRE isolates were assigned as controls.

### 4.5. Detection of Mobile Colistin Resistance (mcr) Gene and Conjugative Ability

Detection of *mcr-1*–*mcr-9* was performed by multiplex PCR using previously published primers and conditions [36,37]. Genomic DNA from a colistin-resistant isolate was extracted using a Mini gDNA Bacteria Kit (Geneaid Biotech, LTD, New Taipei City, Taiwan) and used as the DNA template in PCR. Amplification was performed in a total volume of 25 μL containing 1 μL of template, 1X PCR buffer, 1.5 mM MgCl_2_, 0.2 mM dNTPs, 0.5 μM of each primer, and 1 U of *Taq* polymerase (Vivantis Technologies, Selangor, Malaysia). PCR products were analyzed by agarose gel electrophoresis. Amplicons were purified using a GF-1 PCR Clean-up Kit (Vivantis Technologies Sdn. Bhd, Selangor, Malaysia) and sent to a commercial facility for sequencing (Macrogen Inc., Seoul, Korea).

In order to investigate the transferability of *mcr*, a conjugation experiment was carried out by broth mating method using sodium azide-resistant *E. coli* J53 as a recipient. Cultures of donor and recipient cells were mixed and incubated at 37 °C for 24 h without shaking. Transconjugants were selected on Tryptic Soy Agar supplemented with sodium azide (150 µg/mL) and colistin (2 µg/mL). Conjugation frequency was expressed as the number of transconjugants divided by the number of donor cells. The presence of *mcr* in transconjugant was confirmed by PCR. Colistin MIC values of transconjugants were determined by broth microdilution method. Plasmid incompatibility group was determined by PCR-based replicon typing (PBRT) as previously described [38]. 

### 4.6. Data Analysis 

Data were collected and analyzed using SPSS version 17.0 (SPSS, Chicago, IL, USA). Categorical data were expressed as counts and percentages. The incidence rates of UTIs with MDRE and non-MDRE were calculated by dividing the total number of MDRE or non-MDRE by the total number of patients. Univariate analysis was performed using chi-square or Fisher’s exact test as appropriate for categorical variables. All variables with *p* ≤ 0.1 in univariate analysis were included in a backward stepwise multivariate logistic regression analysis to determine risk factors associated with CA-UTIs caused by MDRE. The results were presented as adjusted odds ratios (aOR) with 95% confidence intervals (CI). A *p* value of <0.05 was considered significant.

## Figures and Tables

**Table 1:** Characteristics of patients with urinary tract infections caused by MDRE and non-MDRE.

Variables	Total Patients *n* = 284	No. of Patients with MDRE *n* = 73	No. of Patients with Non MDRE *n* = 211	Univariate Analysis *p* Value ^a^	Multivariate Logistic Regression Analysis		
					*p* Value ^b^	aOR	95% CI
	*n* (%)	*n* (%)	*n* (%)				
Female gender	251 (88.4)	67 (91.8)	184 (87.2)	0.293			
Age ≤ 30 years	92 (32.4)	21 (22.8)	71 (33.6)	0.442			
Age 31−45 years	53 (18.7)	13 (17.8)	40 (19.0)	0.828			
Age 46−60 years	68 (23.9)	16 (21.9)	52 (24.6)	0.638			
Age > 60 years	71 (25.0)	23 (31.5)	48 (22.7)	0.136			
Living in urban area	161 (56.7)	42 (57.5)	119 (56.4)	0.866			
Education							
Primary school	85 (29.9)	25 (34.2)	60 (28.4)	0.350			
Secondary school	115 (40.5)	30 (41.1)	85 (40.3)	0.903			
College and university or higher	78 (27.5)	16 (21.9)	62 (29.4)	0.218			
Occupation							
Government officer or company employee	69 (24.3)	17 (23.3)	52 (24.6)	0.616			
Labor	58 (20.4)	10 (13.7)	48 (22.7)	0.098	0.213	0.620	0.291–1.317
Farmer	22 (7.7)	11 (15.1)	11 (5.2)	0.007	0.013	3.101	1.272–7.564
Having pets at home (dogs or cats)	84 (29.6)	22 (30.1)	62 (29.4)	0.903			
Raising chicken or duck	10 (3.5)	5 (6.8)	5 (2.4)	0.073	0.277	2.086	0.554–7.859
Drinking water							
tap water	35 (12.3)	8 (11.0)	27 (12.8)	0.681			
filtered water	200 (70.4)	47 (64.4)	153 (72.5)	0.190			
Consumption of undercooked meat ^c^	35 (12.3)	11 (15.1)	24 (11.4)	0.408			
Underlying diseases							
Hypertension	60 (21.1)	19 (26.0)	41 (19.4)	0.234			
Dyslipidemia	36 (12.7)	12 (16.4)	24 (11.4)	0.262			
Diabetes	24 (8.5)	7 (9.6)	17 (8.1)	0.685			
Respiratory disease	8 (2.8)	1 (1.4)	7 (3.3)	0.386			
Antibiotic usage within previous 3 months	32 (11.3)	13 (17.8)	19 (9.0)	0.040	0.062	2.093	0.693–4.546
History of hospitalization within previous 6 months	17 (6.0)	7 (9.6)	10 (4.7)	0.132			

Abbreviations: MDRE, multidrug-resistant Enterobacterales; aOR, adjusted odds ratio; CI, confidence interval. ^a^ *p* value < 0.1 was included in multivariate logistic regression analysis. ^b^ *p* value < 0.05 was considered statistically significant. ^c^ pork or chicken.

**Table 2:** Distribution of Enterobacterales with and without multidrug-resistant phenotypes.

Enterobacterales	No. of MDRE *n* = 77	No. of Non-MDRE Isolates *n* = 232	Total No. of Isolates *n* = 309
	*n* (%)	*n* (%)	*n* (%)
*Escherichia coli*	67 (87)	200 (86.2)	267 (86.4)
*Klebsiella pneumoniae*	7 (9.1)	15 (6.5)	22 (7.1)
*Enterobacter cloacae*	1 (1.3)	10 (4.3)	11 (3.6)
*Proteus* spp.	1 (1.3)	3 (1.3)	4 (1.3)
*Proteus mirabilis*	1 (1.3)	1 (0.4)	2 (0.7)
*Enterobacter aerogenes*	0	1 (0.4)	1 (0.3)
*Citrobacter* spp.	0	1 (0.4)	1 (0.3)
*Salmonella* spp.	0	1 (0.4)	1 (0.3)

Abbreviation: MDRE, multidrug-resistant Enterobacterales.

**Table 3:** Antibiotic resistance rates of MDRE and non-MDRE.

Antibiotics	No. (%) of Resistance Isolates			
	Total (*n* = 309)	MDRE (*n* = 77)	Non-MDRE (*n* = 232)	*p* Value ^a^
ampicillin	207 (67.0)	77 (100)	131 (56.5)	<0.001
amoxicillin/clavulanate	19 (6.1)	5 (6.5)	15 (6.5)	0.891
cephalexin	61 (19.7)	30 (39.0)	32 (13.8)	<0.001
cefuroxime	42 (13.6)	28 (36.4)	14 (6.0)	<0.001
cefotaxime	45 (14.6)	30 (39.0)	16 (6.9)	<0.001
ceftriaxone	43 (13.9)	29 (37.7)	14 (6.0)	<0.001
imipenem	0	0	0	ND
gentamicin	49 (15.9)	44 (57.1)	5 (2.2)	<0.001
amikacin	6 (1.9)	4 (5.2)	2 (0.9)	0.096
cotrimoxazole	101 (32.7)	63 (81.8)	38 (16.4)	<0.001
ciprofloxacin	105 (34.0)	62 (80.5)	43 (18.5)	<0.001
levofloxacin	54 (17.5)	37 (48.1)	17 (7.3)	<0.001
fosfomycin	4 (1.3)	2 (2.6)	2 (0.9)	0.245
nitrofurantoin	12 (3.9)	5 (6.5)	8 (3.4)	0.174

Abbreviation: MDRE, multidrug-resistant Enterobacterales; ND, Not determined. ^a^ Compared between MDRE and non-MDRE.

**Table 4:** Common resistance profiles among MDRE and non-MDRE.

Resistance Profiles	MDRE *n* = 77	Non-MDRE *n* = 232
	*n* (%)	*n* (%)
AMP	-	47 (20.3)
AMP-SXT	-	26 (11.2)
AMP-CIP	-	18 (7.8)
AMP-CN-SXT	9 (11.7)	-
AMP-SXT-CIP	8 (10.4)	-
AMP-CN-SXT-CIP-LEV	8 (10.4)	-
AMP-SXT-CIP-LEV	7 (9.1)	-

Abbreviations: MDRE, multidrug-resistant Enterobacterales; AMP, ampicillin; SXT, trime. thoprim/sulfamethoxazole; CIP, ciprofloxacin; CN, gentamicin and LEV, levofloxacin.

## Data Availability

The data presented in this study are available on request from the corresponding author.

## Supplementary Materials

The following supporting information can be downloaded at: https://www.mdpi.com/article/10.3390/antibiotics11081039/s1, Table S1: Antibiotic resistance profiles of MDRE isolated from urine samples; Table S2: Antibiotic resistance profiles of non-MDRE isolated from urine samples.

## References

[1] Klein R.D., Hultgren S.J. (2020). Urinary tract infections: Microbial pathogenesis, host-pathogen interactions and new treatment strategies. Nat. Rev. Microbiol..

[2] Seymour C.W., Gesten F., Prescott H.C., Friedrich M.E., Iwashyna T.J., Phillips G.S., Lemeshow S., Osborn T., Terry K.M., Levy M.M. (2017). Time to treatment and mortality during mandated emergency care for sepsis. N. Engl. J. Med..

[3] Sugianli A.K., Ginting F., Parwati I., de Jong M.D., van Leth F., Schultsz C. (2021). Antimicrobial resistance among uropathogens in the Asia-Pacific region: A systematic review. JAC Antimicrob. Resist..

[4] Pruetpongpun N., Khawcharoenporn T., Damronglerd P., Suwantarat N., Apisarnthanarak A., Rutjanawech S. (2017). Inappropriate empirical treatment of uncomplicated cystitis in Thai women: Lessons learned. Clin. Infect. Dis..

[5] Gupta K., Hooton T.M., Naber K.G., Wullt B., Colgan R., Miller L.G., Moran G.J., Nicolle L.E., Raz R., Schaeffer A.J. (2011). International clinical practice guidelines for the treatment of acute uncomplicated cystitis and pyelonephritis in women: A 2010 update by the Infectious Diseases Society of America and the European Society for Microbiology and Infectious Diseases. Clin. Infect. Dis..

[6] Lim C., Takahashi E., Hongsuwan M., Wuthiekanun V., Thamlikitkul V., Hinjoy S., Day N.P., Peacock S.J., Limmathurotsakul D. (2016). Epidemiology and burden of multidrug-resistant bacterial infection in a developing country. Elife.

[7] Phodha T., Riewpaiboon A., Malathum K., Coyte P.C. (2019). Excess annual economic burdens from nosocomial infections caused by multi-drug resistant bacteria in Thailand. Expert Rev. Pharm. Outcomes Res..

[8] Troiano G., Messina G., Nante N. (2021). Bacterial lysates (OM-85 BV): A cost-effective proposal in order to contrast antibiotic resistance. J. Prev. Med. Hyg..

[9] Suwantarat N., Carroll K.C. (2016). Epidemiology and molecular characterization of multidrug-resistant Gram-negative bacteria in Southeast Asia. Antimicrob. Resist. Infect. Control.

[10] Chautrakarn S., Khumros W., Phutrakool P. (2021). Self-medication with over-the-counter medicines among the working age population in metropolitan areas of Thailand. Front. Pharmacol..

[11] Jean S.S., Coombs G., Ling T., Balaji V., Rodrigues C., Mikamo H., Kim M.J., Rajasekaram D.G., Mendoza M., Tan T.Y. (2016). Epidemiology and antimicrobial susceptibility profiles of pathogens causing urinary tract infections in the Asia-Pacific region: Results from the Study for Monitoring Antimicrobial Resistance Trends (SMART), 2010–2013. Int. J. Antimicrob. Agents.

[12] National Antimicrobial Resistance Surveillance, Thailand. Antimicrobial Resistance 2000–2020, 2020. http://narst.dmsc.moph.go.th/data/AMR%202000-2020-12M.pdf.

[13] Almomani B.A., Hayajneh W.A., Ayoub A.M., Ababneh M.A., Al Momani M.A. (2018). Clinical patterns, epidemiology and risk factors of community-acquired urinary tract infection caused by extended-spectrum beta-lactamase producers: A prospective hospital case-control study. Infection.

[14] Faine B.A., Harland K.K., Porter B., Liang S.Y., Mohr N. (2015). A clinical decision rule identifies risk factors associated with antimicrobial-resistant urinary pathogens in the emergency department: A retrospective validation study. Ann. Pharmacother..

[15] Savatmorigkorngul S., Poowarattanawiwit P., Sawanyawisuth K., Sittichanbunchaet Y. (2016). Factors associated with extended spectrum beta-lactamase producing *Escherichia coli* in community-acquired urinary tract infection at hospital emergency department, Bangkok, Thailand. Southeast Asian J. Trop. Med. Public Health.

[16] Bunjoungmanee P., Tangsathapornpong A., Kulalert P. (2018). Clinical manifestations and risk factors in urinary tract infection caused by community-acquired extended-spectrum beta-lactamase enzyme producing bacteria in children. Southeast Asian J. Trop. Med. Public Health.

[17] Sangsuwan T., Jariyasoonthornkit K., Jamulitrat S. (2021). Antimicrobial resistance patterns amid community-acquired uropathogens in outpatient settings of a tertiary care hospital in Thailand. Siriraj Med. J..

[18] Manges A.R., Smith S.P., Lau B.J., Nuval C.J., Eisenberg J.N., Dietrich P.S., Riley L.W. (2007). Retail meat consumption and the acquisition of antimicrobial resistant *Escherichia coli* causing urinary tract infections: A case-control study. Foodborne Pathog. Dis..

[19] Khawcharoenporn T., Vasoo S., Singh K. (2013). Urinary tract infections due to multidrug-resistant Enterobacteriaceae: Prevalence and risk factors in a Chicago emergency department. Emerg. Med. Int..

[20] Ben Ayed H., Koubaa M., Hammami F., Marrakchi C., Rekik K., Ben Jemaa T., Maaloul I., Yaich S., Damak J., Ben Jemaa M. (2019). Performance of an easy and simple new scoring model in predicting multidrug-resistant Enterobacteriaceae in community-acquired urinary tract infections. Open Forum Infectious Diseases.

[21] Benaissa E., Belouad E., Mechal Y., Benlahlou Y., Chadli M., Maleb A., Elouennass M. (2021). Multidrug-resistant community-acquired urinary tract infections in a northern region of Morocco: Epidemiology and risk factors. Germs.

[22] Boonyasiri A., Tangkoskul T., Seenama C., Saiyarin J., Tiengrim S., Thamlikitkul V. (2014). Prevalence of antibiotic resistant bacteria in healthy adults, foods, food animals, and the environment in selected areas in Thailand. Pathog. Glob. Health.

[23] Magruder M., Sholi A.N., Gong C., Zhang L., Edusei E., Huang J., Albakry S., Satlin M.J., Westblade L.F., Crawford C. (2019). Gut uropathogen abundance is a risk factor for development of bacteriuria and urinary tract infection. Nat. Commun..

[24] Parikumsil N., Prapasawat W., Siriphap A., Chonsin K., Theethakaew C., Sukolrattanamaetee N., Ratchatanpha D., Siripanichgon K., Suthienkul O. (2017). Virulence factors and molecular epidemiology of uropatho genic *Escherichia coli* isolated from paired urine and rectal swab samples of patients with urinary tract infections in Thailand. Southeast Asian. J. Trop. Med. Public Health.

[25] European Association of Urology, Arnhem, The Netherlands. EAU Guidelines on Urological Infections, 2022. https://uroweb.org/guideline/urological-infections/.

[26] Liu Y.Y., Wang Y., Walsh T.R., Yim L.X., Zhang R., Spencer J., Doi Y., Tian G., Dong B., Huang X. (2016). Emergence of plasmid-mediated colistin resistance mechanism MCR-1 in animals and human beings in China: A microbiological and molecular biological study. Lancet Infect. Dis..

[27] Ling Z., Yin W., Shen Z., Wang Y., Shen J., Walsh T.R. (2020). Epidemiology of mobile colistin resistance genes *mcr-1* to *mcr-9*. J. Antimicrob. Chemother..

[28] Paveenkittiporn W., Kamjumphol W., Ungcharoen R., Kerdsin A. (2021). Whole-genome sequencing of clinically isolated carbapenem-resistant Enterobacterales harboring *mcr* genes in Thailand, 2016–2019. Front. Microbiol..

[29] Luk-In S., Chatsuwan T., Kueakulpattana N., Rirerm U., Wannigama D.L., Plongla R., Lawung R., Pulsrikarn C., Chantaroj S., Chaichana P. (2021). Occurrence of *mcr*-mediated colistin resistance in *Salmonella* clinical isolates in Thailand. Sci. Rep..

[30] Li Y., Dai X., Zeng J., Gao Y., Zhang Z., Zhang L. (2020). Characterization of the global distribution and diversified plasmid reservoirs of the colistin resistance gene *mcr-9*. Sci. Rep..

[31] Khanawapee A., Kerdsin A., Chopjitt P., Boueroy P., Hatrongjit R., Akeda Y., Tomono K., Nuanualsuwan S., Hamada S. (2021). Distribution and molecular characterization of *Escherichia coli* harboring *mcr* genes isolated from slaughtered pigs in Thailand. Microb. Drug Resist..

[32] Rozwandowicz M., Brouwer M.S.M., Fischer J., Wagenaar J.A., Gonzalez-Zorn B., Guerra B., Mevius D.J., Hordijk J. (2018). Plasmids carrying antimicrobial resistance genes in Enterobacteriaceae. J. Antimicrob. Chemother..

[33] Lane D.J., Stackebrandt E., Goodfellow M. (1991). 16S/23S rRNA sequencing. Nucleic Acid Techniques in Bacterial Systematics.

[34] Clinical and Laboratory Standards Institute (2018). Performance Standards for Antimicrobial Susceptibility Testing.

[35] Falagas M.E., Karageorgopoulos D.E. (2008). Pandrug resistance (PDR), extensive drug resistance (XDR), and multidrug resistance (MDR) among Gram-negative bacilli: Need for international harmonization in terminology. Clin. Infect. Dis..

[36] Lescat M., Poirel L., Nordmann P. (2018). Rapid multiplex polymerase chain reaction for detection of *mcr-1* to *mcr-5* genes. Diagn. Microbiol. Infect. Dis..

[37] Borowiak M., Baumann B., Fischer J., Thomas K., Deneke C., Hammerl J.A., Szabo I., Malorny B. (2020). Development of a novel *mcr-6* to *mcr-9* multiplex PCR and assessment of *mcr-1* to *mcr-9* occurrence in colistin-resistant *Salmonella enterica* isolates from environment, feed, animals and food (2011–2018) in Germany. Front. Microbiol..

[38] Carattoli A., Bertini A., Villa L., Falbo V., Hopkins K.L., Threlfall E.J. (2005). Identification of plasmids by PCR-based replicon typing. J. Microbiol. Methods.

