# Supplementary material for: Risk Factors for Community-Acquired Urinary Tract Infections Caused by Multidrug-Resistant Enterobacterales in Thailand
Source: Antibiotics (Basel). 2022 Aug 2;11(8):1039. doi: 10.3390/antibiotics11081039 (PMC9405395; doi:10.3390/antibiotics11081039)
Supplement: Supplementary file 1 [file antibiotics-11-01039-s001.zip › antibiotics-1810414-supplementary.pdf]

Table S1. Antibiotic-resistant profiles of MDRE isolated from urine samples (*n* = 77)

Profiles	*Antibiotic-resistant profiles*	*No. of antibiotics*	*No. of isolates*
1	AMP-CL-CXM-CTX-CRO-AK-CN-SXT-CIP-LEV	10	1
2	AMP-AMC-CL-CXM-CTX-CRO-SXT-CIP-LEV	9	2
3	AMP-CL-CXM-CTX-CRO-CN-SXT-CIP-LEV	9	5
4	AMP-AMC-CL-CXM-CTX-CRO-CN-CIP	8	1
5	AMP-AMC-CL-CXM-CTX-CRO-SXT-CIP	8	1
6	AMP-CL-CXM-CTX-CRO-AK-CIP-LEV	8	1
7	AMP-CL-CXM-CTX-CRO-CN-CIP-LEV	8	2
8	AMP-CL-CXM-CTX-CRO-CN-SXT-CIP	8	3
9	AMP-CL-CXM-CTX-CRO-SXT-CIP-LEV	8	4
10	AMP-CL-CXM-CTX-CRO-CN-CIP	7	2
11	AMP-CL-CXM-CTX-CRO-CN-SXT	7	3
12	AMP-CL-CXM-CTX-CRO-SXT-CIP	7	2
13	AMP-CL-CXM-CTX-SXT-CIP-LEV	7	1
14	AMP-CL-CTX-CRO-CN-SXT-CIP	7	1
15	AMP-AMC-CTX-AK-CN-CIP	6	1
16	AMP-CL-CN-CIP-LEV	5	1
17	AMP-CN-SXT-CIP-LEV	5	8
18	AMP-SXT-CIP-LEV-F	5	1
19	AMP-CRO-SXT-CIP	4	1
20	AMP-AK-CN-SXT	4	1
21	AMP-CN-SXT-CIP	4	1
22	AMP-CN-SXT-F	4	1
23	AMP-CN-CIP-LEV	4	3

24	AMP-SXT-CIP-LEV	4	7
25	AMP-SXT-CIP-FOS	4	1
26	AMP-SXT-CIP-F	4	1
27	AMP-CIP-LEV-FOS	4	1
28	AMP-CN-CIP	3	1
29	AMP-CN-SXT	3	9
30	AMP-SXT-CIP	3	8
31	AMP-SXT-F	3	1
32	AMP-CIP-F	3	1

Abbreviations: MDRE, multidrug-resistant Enterobacterales; AMP, ampicillin; AMC, amoxicillin/clavulanate; CL, cephalexin, cefazolin; CXM, cefuroxime; CTX, cefotaxime; CRO, ceftriaxone; IPM, imipenem; CN, gentamicin; AK, amikacin; SXT, cotrimoxazole; CIP, ciprofloxacin; LEV, levofloxacin; FOS, fosfomycin and F, nitrofurantoin.

Table S2. Antibiotic-resistant profiles of non-MDRE isolated from urine samples (*n* = 232)

Patterns	*Antibiotic-resistant profiles*	*No. of antibiotics*	*No. of isolates*
1	AMP-CL-CXM-CTX-CRO-CIP-LEV	7	2
2	AMP-CL-CXM-CTX-CRO-CIP	6	1
3	AMP-CL-CXM-CTX-CRO-SXT	6	4
4	AMP-AMC-CTX-AK-CN	5	1
5	AMP-AMC-CL-CTX-F	5	1
6	AMP-CL-CXM-CTX-CRO	5	1
7	AMP-CXM-CTX-CRO-CIP	5	1
8	AMP-AMC-CL-CXM	4	2
9	AMP-AMC-CL-FOS	4	1
10	AMP-AMC-CL	3	3
11	AMP-CL-SXT	3	2
12	AMP-CL-CIP	3	1
13	AMC-CL-F	3	1
14	AMP-CRO-SXT	3	1
15	AMP-CIP-LEV	3	7
16	CTX-AK-CN	3	1
17	CN-CIP-LEV	3	2
18	SXT-CIP-LEV	3	1
19	AMP-CL	2	4
20	AMP-SXT	2	26
21	AMP-CIP	2	18
22	AMP-LEV	2	1

23	AMP-FOS	2	1
24	AMP-F	2	2
25	AMC-CL	2	1
26	CL-CXM	2	1
27	CL-CRO	2	1
28	CIP-LEV	2	3
29	AMP	1	47
30	CL	1	2
31	CTX	1	2
32	CRO	1	2
33	CN	1	1
34	SXT	1	3
35	CIP	1	6
36	F	1	1
	Susceptible to all antibiotics tested		77

Abbreviations: MDRE, multidrug-resistant Enterobacterales; AMP, ampicillin; AMC, amoxicillin/clavulanate; CL, cephalexin, cefazolin; CXM, cefuroxime; CTX, cefotaxime; CRO, ceftriaxone; IPM, imipenem; CN, gentamicin; AK, amikacin; SXT, cotrimoxazole; CIP, ciprofloxacin; LEV, levofloxacin; FOS, fosfomycin and F, nitrofurantoin.
